# Prophylactic antibiotics may not be necessary for transoral endoscopic thyroidectomy

**DOI:** 10.3389/fsurg.2022.940391

**Published:** 2022-08-02

**Authors:** Jun Sung Lee, Hee Jun Kim, Jin Seok Lee, Hyeok Jun Yun, Suji Lee, Jae-Ho Cheong, Soo Young Kim, Seok-Mo Kim, Hojin Chang, Yong Sang Lee, Cheong Soo Park, Hang-Seok Chang

**Affiliations:** ^1^ Department of Surgery, Thyroid Cancer Center, Gangnam Severance Hospital, Institute of Refractory Thyroid Cancer, Yonsei University College of Medicine, Seoul, South Korea; ^2^ Department of Surgery, CHA Ilsan Medical Center, Cha University School of Medicine, Goyang-si, South Korea; ^3^ Department of Surgery, Graduate school of medical science, BK21 Project, Yonsei University College of Medicine, Seoul, Korea; ^4^ Department of Surgery, Ajou University School of Medicine, Suwon, Korea

**Keywords:** thyroid cancer, transoral thyroidectomy, culture, antibiotics, surgical site infection

## Abstract

**Background:**

With the recent advances in thyroid cancer surgery techniques and the increasing number of patients concerned about cosmetics, the use of transoral endoscopic thyroidectomy is increasing globally. The aim of this study was to determine whether transoral endoscopic thyroidectomy is truly a clean-contaminated surgery.

**Methods:**

From September 2016 to April 2018, 20 patients with thyroid cancer underwent transoral endoscopic thyroidectomy performed by a single surgeon at Gangnam Severance Hospital. Before and after surgery, the oral cavity was swabbed to obtain culture samples, and antibiotics were administered before and after surgery each once.

**Results:**

Of the total 20 patients, no bacteria were identified before or after surgery in eight (40%) patients. Bacteria were identified both before and after surgery in seven patients (35%). In four patients (20%), bacteria were not identified before surgery, but bacteria were identified after surgery. Bacteria were identified before surgery but not after surgery in one patient (5%). No surgical site infection was observed. All the bacteria identified were normal flora of the oral cavity and skin.

**Conclusions:**

There was no difference between the preoperative culture and postoperative culture of the oral cavity in patients undergoing TOET, and there were no postoperative surgical site infection with prophylactic pre & post-operative antibiotics use. Considering the patient's position and surgical extent in TOET, it appears to be difficult for non-indigenous bacteria to invade the surgical site in oral cavity.

## Background

With the recent development in thyroid cancer surgery and the increasing number of patients concern about cosmetics, the use of transoral endoscopic thyroidectomy (TOET) is spreading globally (1, 2). After the first reports of TOET in 2008 (3), it has become one of the option of surgical treatment for papillary thyroid cancer (PTC).

The TOET approach is considered clean-contaminated surgery because the access through the oral cavity can transfer oral flora into the thyroid space and may increase the risk of surgical site infection (4). At the Gangnam Severance Hospital, we use one dose of flomoxef 1 g preoperatively and one dose of flomoxef 1 g postoperatively as a prophylactic antibiotic. The use of prophylactic antibiotics remains controversial and there are no standard guidelines for the use of antibiotics in TOET (5–8). The aim of this study was to determine whether or not antibiotics are required before and after surgery.

## Methods

From October to December 2019, 20 patients who underwent TOET due to PTC were enrolled in this study. All surgeries were performed by a single surgeon at Gangnam Severance Hospital. Before and after surgery, the oral cavity was swabbed to for testing of bacteria culture, and flomoxef 1 g was administered once before surgery and once after surgery.

**Figure 1:**
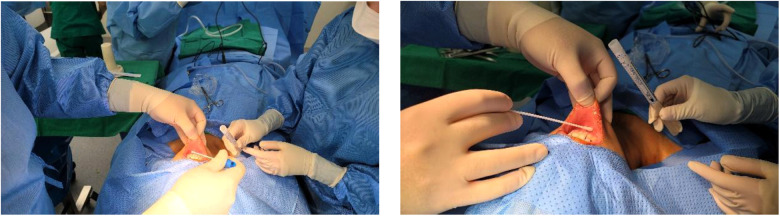
Preoperative culture.

**Figure 2:**
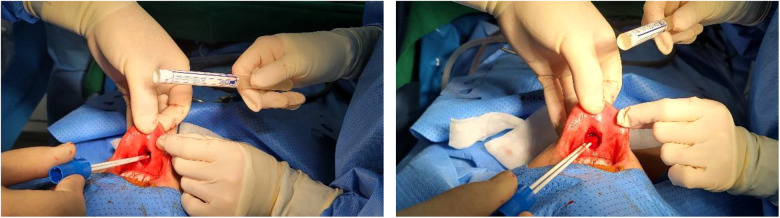
Postoperative culture.

Figures 1, 2 show the swabbing procedure for obtaining the preoperative and postoperative culture at the incision site in the oral cavity. We irrigated oral cavity twice with 50 cc 1:1 saline with providone iodine after endotracheal induction. We used a culture tube including two cotton swabs for obtaining the specimens at the incision site. Swabbing was performed by the operator and first assistant once before surgery and once after surgery. The preoperative culture sample was obtained immediately before the incision was made, and the postoperative culture sample was obtained after the patient was delivered from the operating table and the bleeding was controlled.

The Institutional Review Board of Gangnam Severance Hospital, Yonsei University College of Medicine (Seoul, Korea) approved this study (approval number: 3-2017-0085).

## Results

From October 23, 2019 to December 17, 2019, 20 patients who underwent TOET surgery were enrolled. The patients' characteristics are summarized in Table 1. Nineteen patients were women, and the sex ratio was 19:1. The average BMI was 20.80 ± 3.558 kg/m^2^. Most patients had no medical history, except one patient who had hypertension, two patients who had hyperthyroidism, and one patient who had depression. Two patients underwent total bilateral thyroidectomy, and 18 patients underwent less than total bilateral thyroidectomy. Only one case involved multiplicity, and it was bilateral case. TOET is difficult to perform when there is severe invasion of cancer into adjacent tissue or a necessity for radical lymph node (LN) dissection, and it is impossible to perform modified radical neck dissection using a transoral incision. Given these reasons, the disease stage of all patients who participated in this study was Stage I. Central compartment LN dissection (CCND) was performed in all of cases, and the number of lymph node of CND was 3.8 ± 2.375. A *BRAF* mutation was identified in two patients.

**Table 1:** Patient characteristics.

Variables	Overall Mean ± SD or *n* (%)
Sex	
M	1 (5)
F	19 (95)
Age (years)	33.95 ± 7.352
BMI	20.80 ± 3.558
Smoking Hx	1 (5)
Underlying Dx	
HTN	1 (5)
DM	0 (0)
Hyperthyroidism	2 (10)
Depression	1 (5)
OP type	
Less-than-total	18 (90)
Total	2 (10)
OP time (min)	79.00 ± 21.127
Size	0.695 ± 0.356
Multiplicity	
No	19 (95)
Unilateral	0 (0.0)
Bilateral	1 (5)
Main site	
Right	13 (65)
Left	5 (25)
Isthmus	2 (10)
Tumor site	
Upper	5 (25)
Mid	6 (30)
Lower	7 (35)
Capsular invasion	
No	20 (100)
Yes	0 (0)
Thyroiditis	
No	13 (35)
Yes	7 (65)
CCND	
No	0 (0)
Yes	20 (100)
Total CND	3.8 ± 2.375
Cancer Stage I	20 (100)
*BRAF* mutation	
No	2 (10)
Yes	18 (90)

Abbreviations: BMI, body mass index; CCND, central compartment lymph node dissection; Total CND, Total number of lymph nodes of central compartment lymph node dissection; DM, diabetes mellitus; Dx, diagnosis; HTN, hypertension; Hx, history; OP, operation; SD, standard deviation.

The preoperative and postoperative bacteria culture results are presented in Table 2. Bacteria was detected in eight patients in the preoperative culture, and in 11 patients in the postoperative culture. In seven patients, bacteria were detected in both the preoperative and postoperative culture. In eight patients, there was no bacteria detected neither pre-operatively or postoperatively. Culture was positive in the postoperative culture but not in the preoperative culture in four patients, and there was only one patient with positive postoperative culture and negative preoperative culture. There were no postoperative surgical site infection observed in this study.

**Table 2:** Preoperative and postoperative culture results.

Variables	Overall, *n* (%)
Preoperative culture (+)	
No	12 (60)
Yes	8 (40)
Postoperative culture (+)	
No	9 (45)
Yes	11 (55)
Surgical site infection	
No	20 (100)
Yes	0 (0)
Pre and post culture (−)	8 (40)
Pre and post culture (+)	7 (35)
Post culture only (+)	4 (20)
Pre culture only (+)	1 (5)

Table 3 shows the detail of preoperative and postoperative bacteria culture. In preoperative cultures, there were eight positive culture results in 20 patients. Most of the preoperative cultures detected were gram-positive cocci. *α*-streptococcus was detected in five cultures, and *Streptococcus sanguinis* and *Staphylococcus epidermidis* grew in one and two preoperative cultures, respectively. Gram-positive bacilli (diphtheroids) and anaerobic gram-positive bacilli (*Propionibacterium acnes*) were also detected in one patient each. Eleven postoperative cultures were positive. Similar to the preoperative cultures, the majority of positive results were gram-positive coccus. *α*-streptococcus, *Staphylococcus epidermidis*, and *Streptococcus sanguinis* were detected in four, three, and three cultures, respectively. Anaerobic gram-positive bacilli were also detected in three cultures. No gram-negative bacteria were detected in any cultures.

**Table 3:** Details of preoperative and postoperative bacteria culture.

Unit	Preoperative culture	Postoperative culture
1	G(+) cocci (*α-streptococcus*)	G(+) cocci (*Staphylococcus epidermidis)*
2	Anaerobic G(+) bacilli (*Propionibacterium acnes*), *Staphylococcus epidermidis*)	Anaerobic G(+) bacilli (*Actinomyces sp.*), G(+) cocci (*α-streptococcus*)
3	No growth	No growth
4	No growth	No growth
5	No growth	Anaerobic G(+) bacilli (*Actinomyces naeslundii*)
6	No growth	No growth
7	No growth	Anaerobic G(+) bacilli (*Propionibacterium acnes*)
8	No growth	G(+) cocci (*Streptococcus sanguinis*)
9	No growth	No growth
10	No growth	No growth
11	G(+) cocci (*α-streptococcus*)	G(+) cocci (*Staphylococcus epidermidis*)
12	No growth	No growth
13	G(+) bacilli (diphtheroids)	No growth
14	G(+) cocci (*α-streptococcus*)	G(+) cocci (*α-streptococcus*)
15	No growth	No growth
16	G(+) cocci (*α-streptococcus*)	G(+) cocci (*α-streptococcus*)
17	G(+) cocci (*α-streptococcus*)	G(+) cocci (*Staphylococcus epidermidis*)
18	No growth	G(+) cocci (*Streptococcus sanguinis*)
19	No growth	No growth
20	G(+) cocci (*Streptococcus sanguinis, Staphylococcus epidermidis*)	G(+) cocci (*Streptococcus sanguinis*)

Abbreviation: G(+), gram-positive.

## Discussion

TOET surgery was first reported in 2008 and first performed on cadavers and pigs (3). Since then, it has become a significant choice for patients who regard cosmetic appearance to be important. It results in no skin scarring and requires only a relatively small skin flap in the gingival mucosa. In conventional open thyroidectomy, there is no need for prophylactic antibiotics usage to prevent surgical site infection because the thyroidectomy wound that occurs as a result of the use of an anterior neck approach is a clean wound (9). However, in TOET surgery, the oral cavity is the incision site and this is considered part of the alimentary tract, so the TOET wound is not considered a clean wound, but a clean-contaminated wound (4, 10). Currently, prophylactic antibiotics in TOET are controversial and the prophylactic antibiotics usage for TOET is different depending on medical centers. Usually antibiotics used for 7 days routinely (6, 8), but there have been several literature to have a trial about reducing the amounts of prophylactic antibiotics or not using antibiotics before and after surgery (5, 7). At the Gangnam Severance Hospital, we use flomoxef, a type of third generation cephalosporin, as a prophylactic antibiotic.

In this study, we planned to check preoperative and postoperative surgical site bacteria culture and postoperative surgical site infection. Through analysis of the collected cultures, we aimed to determine if prophylactic antibiotics usage is essential in TOET, and what was the main infection source if surgical site infection occurred. Specimens for bacteria culture were collected twice, once before the incision was made at the gingival mucosa, and once before the incision site was closed. After the surgery, no drain was inserted at the surgical site. Flomoxef 1 g was used preoperatively and postoperatively each once as a prophylactic antibiotic.

The results showed that there was no difference between the preoperative and postoperative culture. In addition, all bacteria detected were normal flora of the oral cavity, and no non-indigenous flora were detected in the oral cavity of any of the patients. There were no postoperative complications, so it was impossible to determine the infection source of any surgical site infections.

These results may indicate that the normal flora function as a barrier (11). The normal bacterial flora of the oral cavity clearly benefit from their host who provides nutrients and habitat. There may also be benefits to the host. The normal flora occupy available colonization sites and this makes it more difficult for non-indigenous species to live in the oral cavity. Moreover, the oral cavity flora also create microbial antagonism against non-indigenous species by production of inhibitory substances such as fatty acids, peroxides, and bacteriocins (12).

The study results show that it is difficult for potential pathogens, such as non-resident bacterial flora, to invade the surgical site of TOET. In this regard, the TOET would have a possibility as a surgery that don't need prophylactic antibiotics because there was no invasion of non-resident bacterial flora but just a normal flora of oral cavity. If further studies are conducted on transoral surgery that is performed without about prophylactic antibiotics, this may lead to less demand for antibiotics for TOET.

There were some limitations in this study. First, the number of patients enrolled in this study was relatively small, thereby reducing the statistical strength of the results. This study can be a preliminary data for the possibility of non-antibiotics need in TOET, but can't play a role as solid evidence because of limitation of the study. Second, the study was retrospective in design which may indicate the presence of selection bias. In addition, antibiotics were used in every patient in this study, so we did not investigate of the rates of surgical site infection when prophylactic antibiotics were not used.

In conclusion, there was no difference between the preoperative culture and postoperative culture of the oral cavity in patients undergoing TOET, and there were no postoperative surgical site infection with prophylactic pre & post-operative antibiotics use. Considering the patient's position and surgical extent in TOET, it appears to be difficult for non-indigenous bacteria to invade the surgical site in oral cavity.

## Data Availability

The original contributions presented in the study are included in the article/Suplementary Material, further inquiries can be directed to the corresponding author/s.
